# Delay in referral of oropharyngeal squamous cell carcinoma to secondary care correlates with a more advanced stage at presentation, and is associated with poorer survival

**DOI:** 10.1038/sj.bjc.6603044

**Published:** 2006-03-21

**Authors:** M Pitchers, C Martin

**Affiliations:** 1 School of Biological Sciences, University of East Anglia, Norwich NR4 7TJ, UK; 2 Department of Oncology, Norfolk and Norwich University Hospital NHS Trust, Norwich, UK

**Keywords:** delay, referral, head and neck cancer

## Abstract

Squamous carcinoma of the oropharynx presents with symptoms common to many benign diseases, and this can cause delay in referral to secondary care. We investigate delay in referral, defining this as the time from symptom-onset to date of general practitioners referral letter to secondary care, and the effect of that delay, using a retrospective case notes based study of patients presenting at our institution with oropharyngeal squamous carcinoma between 1995 and 2005. Using correlation analysis and ordinal regression, we examined the relationship between increased referral delay from primary care, clinical stage at presentation, and survival. Increasing time from symptom onset to referral to secondary care was positively correlated with more advanced disease stage at presentation (*r*_s_=+0.346, *P*=0.004). This was confirmed with ordinal regression modelling (delay estimate=0.045, *P*=0.042). Patients with delay of less than 6 weeks had significantly improved survival compared to those with a delay of greater than 6 weeks (*P*=0.032). For every 1 week of delay in referral, we estimate that the stage of presentation will progress by 0.045 of ‘a stage’.

Head and neck squamous cell carcinoma (HNSCC) comprises 3% of new cancer diagnoses per year in the UK (BAO-HNS, 2002). Oropharyngeal HNSCC comprises mainly tumours of the base of tongue, pharyngeal tonsils, soft palate, and uvula. Less common sites are posterior pharyngeal wall, tonsillar pillars, and glossotonsillar sulci. Oropharyngeal HNSCC has an incidence of 1.7 : 100 000 in the Eastern region of the UK with a male to female ratio of 2.6 : 0.8 (ECRIC, 1999). The disease shows notable geographic variation: for example, the Calvados region in France has an incidence of 13.9 : 100 000 (Peckham *et al*, 1995). Many patients present with nonspecific symptoms, such as sore throat or neck nodes. Five-year survival rates are related to stage at presentation, stage I tumours having a 67% 5-year survival, stage IV 15–19% (Peckham *et al*, 1995). The majority of patients tend to present in advanced stage, with a worse prognosis (Dhooge *et al*, 1996).

Owing to the relative rarity of the tumour, the average general practitioner (GP) is not likely to see more than one case in his professional lifetime. Thus, the index of suspicion is low. By contrast the presenting symptoms – generally sore throat and/or neck nodes – are common, nonspecific and shared with commoner benign conditions such as viral pharyngitis or tonsillitis. Thus, many patients are treated by courses of antibiotics for weeks or months causing a delay in referral to secondary care, generally to an ENT specialist.

Delays in the referral of these patients to secondary care are a problem worldwide. Four papers have examined the delay from symptom-onset to various times in the care pathway. Scully *et al* (1986) found a mean delay of 3.5 months from first symptom awareness to referral from primary care in oral cancer patients, although Onizawa *et al* (2003), also in oral cancer patients, found a mean of 2.7 months to diagnosis. Jones *et al* (2002) in head and neck cancer patients found a mean 4.9 months to presentation at secondary care. Amir *et al* (1999) studied within-patient-delay specifically and found a mean 7.4 weeks for oral cancer patients and 12.3 weeks for other head and neck cancers. None of these authors analysed the relation between delay and tumour stage or patient survival.

For clarity, delays in the referral and treatment pathway can be divided into five stages: (1) Patient delay in seeking medical advice (within-patient delay). (2) Primary care delay in referral to a specialist up till the date of GP referral (within-GP delay), that is, time from first presentation to GP with symptom to date of referral letter. (3) Delay from referral letter to time of specialist appointment. (4) Delay from specialist appointment to results of investigations (principally endoscopy, histology, and scans). (5) Delay from results being available to start of treatment.

In the UK, referrals of suspected new cases of cancer from primary care to a specialist have been subject to a ‘2-week rule’ since the year 2000 (NHS Executive, 1999) (Appendix A). All suspected cancer cases are required to be seen within 2 weeks of referral from their GP. Since 2000, there has also been improved national funding of oncology services and two new targets which have been recently introduced: a 1-month target from decision on treatment to start of treatment and a two-month target from GP referral (in urgent cases) to treatment (Great Britain, Department of Health, 2000). Since the year 2000 is the median year of our study period, the time from GP referral to start of treatment is likely to be much shorter in the period 2000–2005 than it was in 1995–2000. As far as the patient is concerned, the crucial period is the overall time from start of symptoms to start of treatment, but we felt that the analysis of this might be biased by changes in the period from GP referral to treatment (stages 3, 4, and 5 in the delay pathway above) in the latter 5 years. Apart from this, the within-patient delay plus the within-GP delay (stages 1 and 2 in the delay pathway as above) make up the predominant part of the overall delay.

For these reasons, we have concentrated on the sum of the first two stages of delay in this study. We have called this period ‘delay in referral’ hereafter in this paper and have analysed it relation to tumour stage and patient survival.

## METHODS

**Figure 1:**
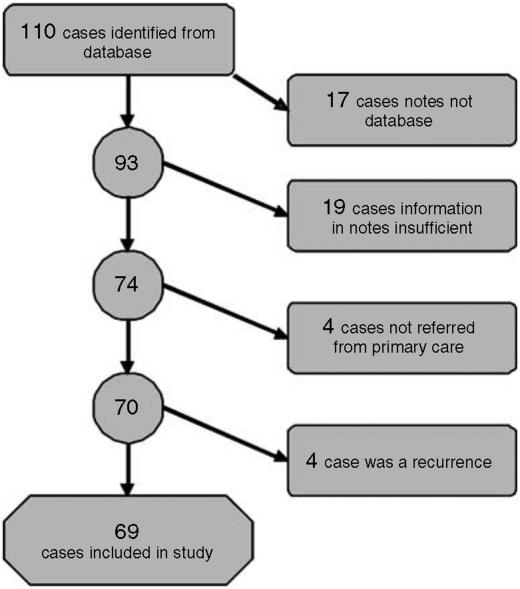
Consort diagram for case selection.

This was a retrospective case notes-based study covering the 10 years from June 1995 to June 2005. Patients were identified from the Department of Oncology computerised database. This produced 110 cases of squamous carcinoma of the oropharynx, the notes of whom were examined and the following information searched for age, gender, site of primary cancer, date of symptom onset, date of referral from primary care, TNM stage at presentation, presenting symptom(s), any treatment given in primary care (to see if treatment given in primary care influenced delay), and outcome. Sources for the data were referral letter from primary care, case notes from first outpatient consultation usually ENT, and oncology notes. In 19 cases, the required information could not be reliably sourced in the notes (see below) and in a further 17 cases the notes could not be located and these patients were excluded. A further four cases were excluded because the referral was not from primary care (since we wished to study only those cases referred by the primary care-secondary care route), and a further one for whom the cancer was a recurrence, leaving 69 cases for analysis. Two cases had incomplete staging data and were excluded from staging analysis. A consort diagram shows this schematically (Figure 1).

In order to extract the data on delay in referral for each patient as precisely as possible, it was necessary to find the time-difference between the date of first symptom and the date of GP's letter referring patient to secondary care. The latter date was easy to find in the patient's notes, but the former was more problematic. If the GP's letter stated clearly the time, for example, ‘patient complains of sore throat for past 6 weeks’ or ‘since beginning of April’, this time was noted. If the GP's letter was vague on this issue, then the ENT records were searched and if this gave the date of first symptom, this was noted. If the ENT record also failed to give the date, then the oncology record was searched (this was uncommon) and its date used if it was named precisely. If none of the GP letter, ENT (or other secondary care) or the oncology record gave reliable information, the patient was excluded.

The data for delay in referral was right skewed, and not normally distributed (visual inspection of histogram and Shapiro–Wilk test); therefore, a Naperian log (ln) transformation was applied, and this gave a normal distribution. To test for differences between groups, the independent samples *t*-test (for two grouping variables) and one-way ANOVA (for three grouping variables) were used, on the ln transformed data. Spearman's rank correlation and PLUM ordinal regression were used to check for any association between delay in referral and stage at presentation, which are nonparametric tests and could be used on the original data. Outcome (survival) was assessed using Kaplan–Meier survival analysis, and the log-rank test. Statistical analysis was carried out using SPSS v12.0® (SPSS Inc., Chicago, USA). Graphs were prepared using SPSS® and GraphPad Prism v4.3® (GraphPad software, San Diego, USA), both for Microsoft Windows XP®.

Statistical advice was given by Econometrics Department at University of East Anglia.

## RESULTS

In the sample group, the average age at presentation was 57.5 (range 38–81, SE of mean 1.46), 54 were male, 15 female. The male to female ratio was 3.6 : 1. Six patients presented with stage II tumours, 16 with stage III, 35 with stage IVA, and 10 with stage IVB. No stage I or stage IVC tumours were identified. With regard to site, 43 were tonsil tumours, 19 posterior tongue, and seven other sites (uvula and palate).

Data were analysed with reference to factors recorded from the case notes. No significant difference was detected with regard to gender, tumour site, presenting symptom, or treatment given in primary care. Frequencies of presenting symptoms were (%): neck lump (49.3), sore throat (33.3), direct visualisation (5.9), incidental finding at medical appointment (2.9), otalgia (2.9), dysphagia (2.9), and globus symptoms (1.4).

**Figure 2:**
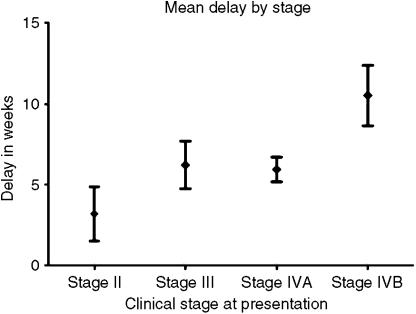
Mean delay (in weeks) by clinical stage at presentation (error bars represent 95% confidence interval of mean).

We examined the relationship between delay in referral and stage at presentation (Figure 2). Although stage is an ordinal variable, it is representative of an underlying trend, so a stage IVA tumour can be considered to have progressed from a Stage III. In order to examine the correlation between delay in referral and stage at presentation, since delay in referral was not normally distributed, and stage is an ordinal variable, we used Spearman's rank correlation (Table 1). This gave a correlation coefficient (*r*_s_) of +0.309, which when *n*=67, gives a two-tailed *P*-value of 0.011, that is significant. Therefore, there is a positive relationship (not necessarily linear) between increased delay in referral and more advanced stage at presentation. To examine this further, we performed an ordinal regression analysis using PLUM (PoLytomous Universal Model). Since the distribution of the delay in referral is right-skewed the negative log–log is the appropriate link function (SPSS website). The model fitting information shows that the null hypothesis: that all the variable coefficients are equal to zero is false (*P*=0.028). The goodness of fit tests are greater than 0.05, indicating that the model fits the data well. Parameter estimates showed the estimate for delay in referral to be 0.045 and the corresponding significance test gave *P*=0.042. Thus, there is a positive relationship between increased delay in referral and advanced stage at presentation, significant at the 0.05 level. Put another way, for every 1-week increase in delay in referral, it is estimated that the stage at presentation will progress by 0.045 of ‘a stage’. Mean referral times by stage are shown (Figure 2).

**Figure 3:**
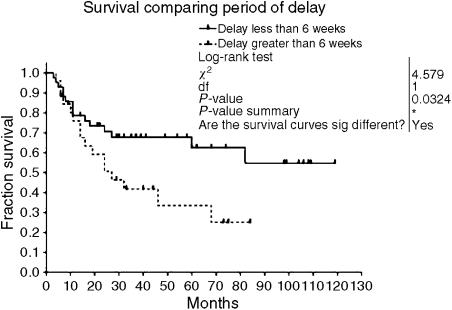
Kaplan–Meier survival analysis of patients with a greater than 6 weeks delay in referral compared to patients with less than 6 weeks delay.

In order to test the possibility that delay in referral might affect outcome, we performed survival analysis comparing two groups with different durations of delay in referral (6 weeks or less, compared to greater than 6 weeks). Six weeks was chosen as a cutoff point, since this was felt to be a typical guide time from the onset of symptoms to the decision to refer. Kaplan–Meier survival curves are shown in Figure 3. Log-rank test showed that the group with less than 6 weeks delay had significantly better survival (*P*=0.032). In our study, proportional hazards for both groups are assumed, as there is no difference in management policy for each group.

## DISCUSSION

Our results show that there is a significant relationship between delay from onset of symptoms to referral to a specialist centre, and stage at presentation. As presenting stage affects overall survival, this delay will be expected to impact on patient outcome, and this is reflected in our survival data. As many of the initial symptoms of oropharyngeal HNSCC are nonspecific, patients may delay in seeking advice from their GP. When presenting symptoms are more specific or worrying to the patient, such as painful ulceration or bleeding, presentation tends to be earlier (Kowalski *et al*, 1994). When nonspecific symptoms are due to minor illness they are usually of limited duration, but persistence of symptoms may indicate more sinister pathology. Delay in diagnosis is one of the most common reasons for litigation in HNSCC (Lydiatt, 2002, 2004).

The retrospective case note based design of the study introduces a potential for inaccuracy, since we are solely reliant on the data available in the notes. As described above, cases from whose notes a reasonably precise date of first symptom could not be extrapolated were excluded. Even cases whose notes said ‘a few weeks’ were excluded, since this could mean anywhere from 2 to 6 weeks. If the notes said, for example, ‘4 months’, this was taken as 17 weeks, although there is an inherent inaccuracy since the time could have been from 3.5 to 4.5 months. We regard our data as accurate to about 2 weeks. A prospective patient questionnaire-based study would give more accurate information but would take many years to complete. We therefore regard our work as a pilot, which can identify a potential area of concern for future study.

Previous studies examining delay in referral and diagnosis of oral and oropharyngeal carcinoma have not shown a correlation between delay and more advanced stage at presentation (Robinson *et al*, 1984; Guggenheimer *et al*, 1989; Merletti *et al*, 1990; Jovanovic *et al*, 1992; Gorsky and Dayan, 1995; Dhooge *et al*, 1996; Hollows *et al*, 2000; Onizawa *et al*, 2003). However, none of these authors concentrated on the within-patient delay or the within-primary care delay, whose sum is likely to be longer than the time from specialist referral to starting treatment. Ours is the first study to analyse delay from symptom onset to specialist referral in this particular type of tumour, which tends to present late, and may explain why our findings are positive while others are not.

Other factors are important in tumour stage at presentation, such as biological behaviour, and some have proposed in other tumour sites that this is more important than delay (Symonds *et al*, 2000). One study examining delay to treatment in endometrial cancer in fact showed an inverse relationship between delay and survival (Crawford *et al*, 2002). This may be due to more biologically aggressive tumours (those with shorter cell cycle time) being seen and treated earlier, yet still having a worse prognosis, but these authors did not look at the time from the first symptom.

Finally, since reducing delay in referral should result in these tumours being seen in secondary care at an earlier stage, there should be financial implications. Oropharyngeal tumours in stages I–II are generally given single-modality, but stages III–IV multimodality, treatment. Also, patients in stages in stages III–IV are more likely to relapse and require palliative care. For both of these reasons reducing delay in referral by increasing patient and GP awareness of this disease should reduce health costs, as well as saving patients' lives.

## CONCLUSIONS

Our study in oropharyngeal squamous carcinoma shows a positive association between delay in referral, more advanced stage at first presentation, and shorter survival. While other factors such as tumour biology also determine stage at presentation, more national efforts should be made to reduce delay in referral.

## Figures and Tables

**Table 1:** Correlations for delay *vs* stage at presentation

			**Delay (weeks)**	**Clinical stage at presentation**
Spearman's rho	Delay (weeks)	Correlation coefficient	1.000	0.309 (*)
		Sig. (two-tailed)		0.011
		*N*	67	67

* Correlation is significant at the 0.05 level (two-tailed).

## Appendix A

Department of Health, November 1999.

### GUIDELINES FOR URGENT REFERRAL OF PATIENTS WITH SUSPECTED CANCER

#### Head and neck cancer

- Ulceration of oral mucosa persisting for >2–3 weeks
- Hoarseness persisting for >2–3 weeks
- Dysphagia persisting for 2–3 weeks
- Unresolving neck masses for 2–3 weeks
- Cranial neuropathies
- Orbital masses

The level of suspicion is further increased if the patient is a heavy smoker or heavy alcohol drinker, and is aged over 45 years.
